# Mechanisms of interleukin 4 mediated increase in efficacy of vaccines against opioid use disorders

**DOI:** 10.1038/s41541-020-00247-7

**Published:** 2020-10-21

**Authors:** Bethany Crouse, Christine Robinson, April Huseby Kelcher, Megan Laudenbach, Juan E. Abrahante, Marco Pravetoni

**Affiliations:** 1 Department of Pharmacology, University of Minnesota Medical School, Minneapolis, MN 55455 USA; 2 Department of Veterinary Population Medicine, University of Minnesota, St. Paul, MN 55455 USA; 3 Department of Psychiatry and Behavioral Sciences, University of Minnesota Medical School, Minneapolis, MN 55455 USA; 4 Hennepin Healthcare Research Institute, Minneapolis, MN 55404 USA; 5 University of Minnesota Informatics Institute, Minneapolis, MN 55455 USA; 6 Center for Immunology, University of Minnesota, Minneapolis, MN 55455 USA

**Keywords:** Adjuvants, Conjugate vaccines, Addiction

## Abstract

Opioid use disorders (OUD) affect over 27 million people worldwide. Anti-opioid vaccines offer a promising strategy to treat OUD and prevent overdose. Using immunomodulation of cytokine signaling to increase vaccine efficacy, this study found that blocking IL-4 improved the efficacy of vaccines targeting oxycodone and fentanyl in male and female mice. Genetic deletion of the IL-4 receptor, STAT6, or antibody-based depletion of IL-13, did not increase vaccine efficacy against opioids, suggesting the involvement of type I IL-4 receptors. Enhancement of vaccine efficacy with blockade of IL-4 was associated with improved germinal center formation in secondary lymphoid organs and selective transcriptome signatures in the activated CD4^+^ T cell population subset. These data suggest that IL-4 is both a pharmacological target and a potential biomarker of vaccine efficacy against OUD.

## Introduction

Opioid use disorders (OUD) affect over 2 million people in the United States and 27 million people worldwide^1,2^. In 2016, 42,200 deaths occurred due to opioid misuse in the US, and up to 81,700 fatal overdoses per year are projected by 2025^3^. Although approved medication assisted treatment (MAT) consisting of pharmacological agonists and antagonists of the opioid receptors is available, these medications display suboptimal clinical efficacy due to side effects, regulatory hurdles that limit patient access, and the potential for diversion and abuse^4–8^. As an alternative or complementary option to MAT, anti-opioid vaccines are an emerging strategy to counteract OUD and overdose^9–11^. Active immunization with opioid-based small molecule haptens conjugated to immunogenic carriers elicit production of drug-specific polyclonal antibodies, which selectively bind to the target drug in the blood and prevent its distribution to the brain^12–15^. Preclinical studies have provided proof of efficacy, selectivity, and safety for this approach to reduce drug-seeking behavior and to prevent respiratory depression, bradycardia, and fatal overdose^14,16–20^. Although the efficacy of anti-opioid vaccines has not been tested in humans, clinical trials for vaccines against cocaine and nicotine showed that a subset of immunized patients produced sufficient levels of high affinity serum IgG antibodies required to achieve vaccine efficacy against nicotine and cocaine^21–23^. These data suggest that vaccine efficacy against drugs of abuse is attainable, but that our understanding of the immunological mechanisms underlying anti-drug antibody responses needs to be further explored. To support the translation of anti-opioid vaccines, efforts are needed to draft a rational blueprint for vaccine development by further understanding the immunological mechanisms underlying optimal vaccine-induced antibody responses against drugs of abuse.

To date, the immunological mechanisms of vaccines against OUD and substance use disorder (SUD) are relatively understudied. We have previously demonstrated that generation of effective anti-opioid antibodies requires CD4^+^ T cell dependent B cell activation by showing that depletion of CD4^+^ T cells or immunization of T cell receptor α (TCRα) subunit knockout mice blunted antibody responses and vaccine efficacy against opioids^15,24^. Analysis of B cell subsets after vaccination showed that early germinal center (GC) formation correlated with antibody titers and vaccine efficacy against oxycodone and nicotine^24,25^. Generally, GC formation involves the activation of B cells and cognate CD4^+^ T cells within the spleen or lymph node follicles, which is mediated by antigen presenting cells (APCs) such as dendritic cells, macrophages, or B cells. Within the GC microenvironment, antigen-specific B cells undergo clonal expansion and affinity maturation to increase antibody specificity for the target antigen^26^. Depending upon molecular signals such as cytokines and co-stimulatory molecules (e.g., CD40), B cells can also undergo class switching recombination of their constant antibody fragments (F_c_), which results in different antibody effector functions^26^. Within the GC, CD4^+^ T helper (Th) cells will also differentiate into Th_1_, Th_2_, Th_17_, T follicular helper (T_fh_), and GC-T_fh_ subsets which coordinate B cell responses to different antigens^27,28^. In this context, B and T cell responses to vaccines can be shaped by the incorporation of adjuvants to achieve successful immunization^29,30^. Pre-clinical studies have already shown that the efficacy of vaccines against SUD is enhanced by adjuvants such as aluminum salts (alum) and toll-like receptor 4 (TLR4) or TLR9 agonists^31–34^. In contrast to more traditional adjuvants, this study focuses on the use of cytokine-targeting immunomodulators as a strategy to enhance GC formation and to increase vaccine efficacy against drugs of abuse.

Our team previously tested whether the efficacy of a candidate vaccine against oxycodone was enhanced by immunomodulators of IL-4, IL-7R (CD127), IL-2R (CD25), programmed death-ligand 1 (PD-L1), or inducible T cell co-stimulator ligand (ICOSL), some of which have been used or tested clinically for other indications^35–39^. This screening strategy found that administration of a neutralizing anti-IL-4 monoclonal antibody (αIL-4 mAb), but not a non-neutralizing αIL-4 mAb^40^, increased the efficacy of a vaccine against oxycodone in mice. This effect was replicated in IL-4^−/−^ mice^40^. The increased efficacy was associated with class switching from IgG_1_ to IgG_2a_ and IgG_3_ and differentiation of CD4^+^ T cells toward T_fh_ and GC-T_fh_ cell subsets^40^, which are needed for GC formation and B cell activation^27^. This is consistent with the hypothesis that the efficacy of anti-opioid vaccines relies primarily on generating high levels of high affinity opioid-specific antibodies. Treatment with an αIL-4 mAb also improved responses to a tetanus toxoid, diphtheria, acellular pertussis (TDaP) vaccine^40^, further supporting this approach for increasing efficacy of other recombinant protein or conjugate vaccines. These data support the hypothesis that IL-4 is both a pharmacological target to increase vaccine efficacy and a biomarker to enhance and predict vaccine efficacy against opioids and other targets, warranting more studies of the IL-4 biology in the context of anti-drug vaccines.

The IL-4 signaling pathway consists of type I and type II receptor complexes. The type I receptor complex involves the IL-4 receptor (IL-4R) binding to the common gamma chain (γ_c_), which signals only in response to IL-4. The type II receptor consists of a complex of the IL-4R and IL-13 receptor (IL-13R), which can be activated by either IL-4 or IL-13. Type I receptors are expressed on most immune cells, while type II receptors are expressed on limited immune cell subsets of myeloid origin and several non-hematopoietic cell populations, including fibroblasts and epithelial cells^41^. The IL-4 signaling pathway is involved in early GC formation in secondary lymphoid organs^42–44^, antibody class switching^45,46^, Th_2_ differentiation^47^, macrophage activation^48^, and mucosal/allergic responses^49^. Although the source of IL-4 during GC formation is still debated, it has been shown that natural killer T cells (NKT cells) and CD4^+^ T cells produce IL-4 after vaccination^42,43,50,51^. Previous literature suggests that IL-4 is involved in several processes within GC formation, including mediating the transition from centroblasts to centrocytes^42^, class-switching^46^, and memory cell differentiation^52^. In contrast, IL-13’s role in GC formation is largely unexplored. IL-4 has also been shown to inhibit GC formation in some contexts^42,50,53^, demonstrating that the role of IL-4 may vary according to the nature or class of antigenic stimuli^40,42–44,53–56^. Therefore, the seemingly pleiotropic role of IL-4 during GC formation is not yet fully uncovered, and its role in modulating the efficacy of conjugate vaccines and antibody responses against small molecules is unknown. Furthermore, the relative contribution of type I vs. type II receptors and the role of IL-13 in mediating the efficacy of anti-drug vaccines has not been elucidated.

This study extended our previous findings that blockade of IL-4 increases the efficacy of anti-opioid vaccines, which generalized to different opioid conjugate vaccine formulations containing structurally different haptens and carrier proteins, and occurred in both males and females. To further dissect this mechanism, we tested the effect of depletion or deletion of downstream signaling components on vaccine efficacy against oxycodone. Surprisingly, we did not see enhanced efficacy upon deletion of IL-4R or its downstream effector, signal transducer and activator of transcription 6 (STAT6). Depletion of IL-13 also did not change vaccine efficacy, suggesting that while IL-4 and its type I receptors were implicated in the increase in vaccine efficacy, IL-13 and type II IL-4 receptors were not. The effect of IL-4 depletion on vaccine efficacy was associated with enhanced GC formation, and was supported by changes in transcriptome signatures found in activated CD4^+^ T cells. In summary, these studies support the use of molecular adjuvants targeting IL-4 to improve vaccine efficacy against opioids and potentially other drugs of abuse, as well as the use of IL-4 as a biomarker of vaccine efficacy for patient stratification or in personalized therapy against OUD or SUD.

## Results

### Neutralization of IL-4 enhances anti-opioid antibody responses in male and female mice

**Fig. 1:**
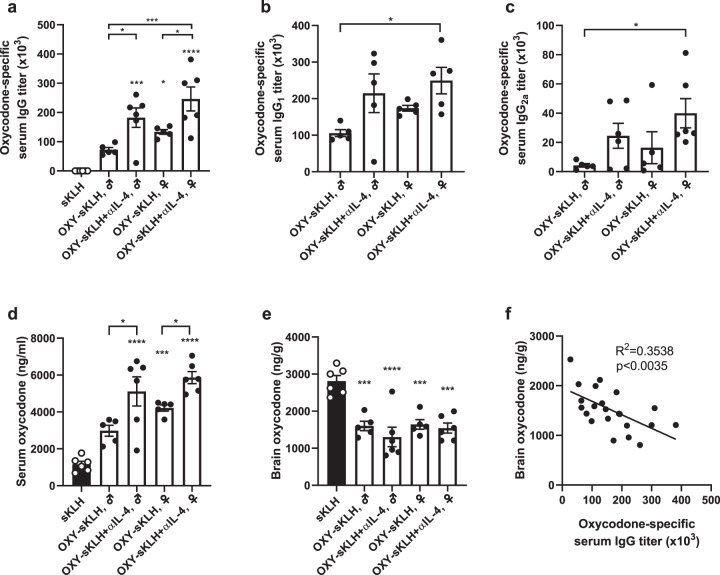
Effect of IL-4 depletion on anti-oxycodone vaccines in male and female mice. Male and female Balb/c mice were immunized on days 0, 14, and 28. (**a**) Oxycodone-specific serum IgG antibody titers on day 34. Oxycodone-specific (**b**) IgG_1_ and (**c**) IgG_2a_ subclass titers. After challenge with 5.0 mg/kg oxycodone, shown: (**d**) serum and (**e**) brain concentrations. (**f**) Relationship between oxycodone-specific serum IgG antibody titers and brain oxycodone concentrations in vaccinated mice. Data are mean ± SEM. Sample size: *n* = 5–6 per group. Statistical analysis conducted by one-way ANOVA paired with Tukey’s multiple comparisons post hoc test (**a**–**e**) or linear regression (**f**). Symbols: *p ≤ 0.05, **p ≤ 0.01, ***p ≤ 0.001, ****p ≤ 0.0001 compared to control, or brackets to indicate differences between groups.

Previous studies showed that concurrent administration of an IL-4 depleting monoclonal antibody (αIL-4) with an anti-oxycodone vaccine increased concentrations of antibody-bound oxycodone in serum, decreased oxycodone concentration in the brain, and resulted in greater efficacy in reducing oxycodone-induced behavioral effects^40^. The increased vaccine efficacy against oxycodone was correlated with higher oxycodone-specific IgG antibody titers and their class switching from IgG_1_ to IgG_2a_ in male Balb/c and C57Bl/6 mice^40^. In a first experiment, these previous findings were expanded by testing the effect of αIL-4 administration in male and female mice. Since the effect of αIL-4 administration was previously established with anti-oxycodone vaccines^40^, a lead anti-oxycodone vaccine consisting of an oxycodone-based hapten (OXY) conjugated to subunit keyhole limpet hemocyanin (sKLH) or native KLH was used as a model for these studies. Both OXY-KLH and OXY-sKLH are effective in preventing oxycodone-distribution to the brain, and reducing oxycodone-induced antinociception, motor activity, respiratory depression and bradycardia, as well as acquisition of oxycodone intravenous self-administration^15–17,34,57^. While oxycodone-specific IgG titers were increased in female mice shortly after the first immunization compared to male mice (Day 14, Supplementary Fig. 1), no differences were found after the third and last immunization (Fig. 1a). Co-administration of αIL-4 with OXY-sKLH increased both IgG_1_ and IgG_2a_ compared to the vaccine alone in both male and female mice at 34 days (Fig. 1b, c). When challenged with oxycodone (5.0 mg/kg, s.c), significant increases in serum oxycodone concentrations were found in both male and female mice treated with αIL-4 compared to mice receiving the vaccine alone (Fig. 1d). These data suggest that IL-4-based mechanisms of vaccine enhancement are conserved between sexes. While brain oxycodone concentrations were reduced in all groups compared to the control (Fig. 1e), no significant differences in brain concentrations were found across treatment groups. In contrast to serum (Fig. 1d), the lack of differences in the efficacy of various vaccine formulations in reducing drug distribution to the brain may be related to the respective pharmacokinetics of unbound (free) oxycodone and antibody-bound oxycodone. For instance, serum opioid-specific antibodies are expected to bind free drug, which prolongs the opioid’s half-life and protects it from metabolism or clearance. This preserves differences in serum drug concentration across treatment groups, which may not be as evident in the brain. Higher individual serum IgG antibody titers were significantly correlated with decreased brain oxycodone concentrations (Fig. 1f), indicating that titers may be used as immune correlates of vaccine efficacy in lieu of significant differences in brain opioid concentration. In fact, it has been previously shown that oxycodone-specific serum IgG antibody titers are predictive of vaccine efficacy against oxycodone in mice and rats^24,25,40^. Significant correlations were also found between antibody titers vs. serum concentrations, as well as brain concentrations vs. serum concentration, indicating that serum oxycodone concentration is also a parameter of vaccine efficacy (Supplementary Fig. 1). Future studies may address the interplay of sex and genetic polymorphisms related to IL-4 signaling in the context of vaccine efficacy.

### Deletion of STAT6 or the IL-4R does not increase vaccine efficacy against opioids

**Fig. 2:**
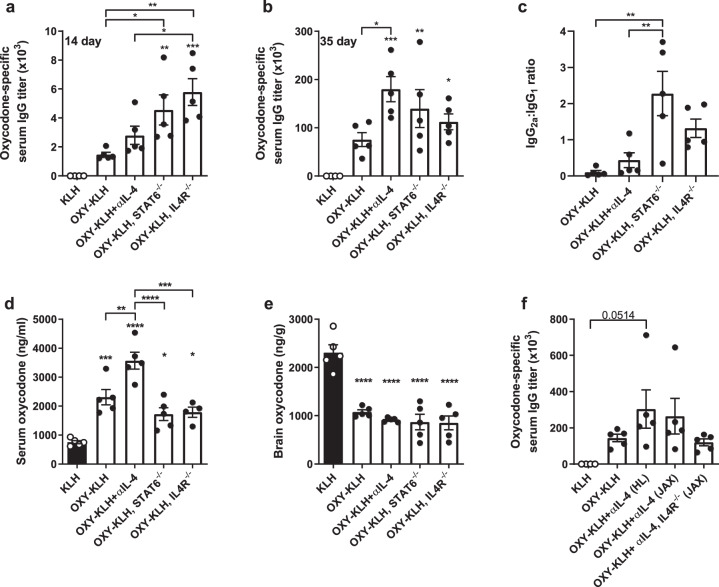
Deletion of downstream IL-4 signaling does not increase efficacy of an anti-oxycodone vaccine. Mice were immunized with either KLH or OXY-KLH on days 0, 14, and 28 and challenged s.c. with 2.25 mg/kg oxycodone on day 35. Oxycodone-specific antibody titers expressed as: (**a**) IgG at 14 days, (**b**) IgG at 35 days, and (**c**) ratio of IgG_2a_ to IgG_1_. At 30 min post-drug challenge, concentration of oxycodone in (**d**) serum and (**e**) brain. (**f**) In a separate experiment, IL4R^−/−^ mice were vaccinated with OXY-KLH and αIL-4, and controls included mice from Harlan (HL) and Jackson (JAX) laboratories. Data are mean ± SEM. Sample size: *n* = 5 per group. Statistical analysis conducted by one-way ANOVA paired with Tukey’s multiple comparisons post hoc test. Symbols: *p ≤ 0.05, **p ≤ 0.01, ***p ≤ 0.001, ****p ≤ 0.0001 compared to control, or brackets to indicate differences between groups.

Because pharmacological or genetic ablation of IL-4 increases vaccine efficacy against opioids^40^, this study further tested whether genetic ablation of individual downstream signaling components could recapitulate this effect. Canonical IL-4 signaling occurs through the IL-4R, which leads to phosphorylation of JAK1/3 and downstream phosphorylation of either STAT6 or insulin receptor substrates (IRS)^41^. Here, IL-4R^−/−^ and STAT6^−/−^ mice were immunized with OXY-KLH and compared to wild-type mice receiving OXY-KLH plus αIL-4. Despite an initial increase in oxycodone-specific serum IgG titers after the first immunization in IL-4R^−/−^ and STAT6^−/−^ mice (Fig. 2a), antibody levels were below those of mice receiving OXY-KLH and αIL-4 after the third vaccination (Fig. 2b). Immunizing IL-4R^−/−^ and STAT6^−/−^ mice with OXY-KLH did not yield an IgG_2a_ to IgG_1_ response ratio equivalent to wild-type mice immunized with OXY-KLH and αIL-4 (Fig. 2c). When challenged with oxycodone, mice receiving OXY-KLH plus αIL-4 showed an increased serum oxycodone concentration compared to the OXY-KLH, indicating higher efficacy, while STAT6^−/−^ and IL-4R^−/−^ mice did not (Fig. 2d). Brain oxycodone levels were significantly decreased in all groups compared to control (Fig. 2e).

These results suggest that while IL-4 depletion increases vaccine efficacy, lack of functional IL-4R or STAT6 does not. To further explain the relationship between blocking IL-4 and IL-4R ablation, we administered OXY-KLH plus αIL-4 to IL-4R^−/−^ mice. To ensure that potential differences were not due to genetic or behavioral differences, wild-type mice from different vendors were included as a control (Fig. 2f). In this experiment, wild-type mice responded similarly to the combination of OXY-KLH and αIL-4, regardless of supplier. However, the combination of OXY-KLH and αIL-4 in IL-4R^−/−^ mice did not yield increased IgG titers compared to wild-type mice receiving OXY-KLH and αIL-4, suggesting that the IL-4R is necessary for the increased efficacy associated with depletion of IL-4.

### Blockage of IL-13 does not increase vaccine efficacy

**Fig. 3:**
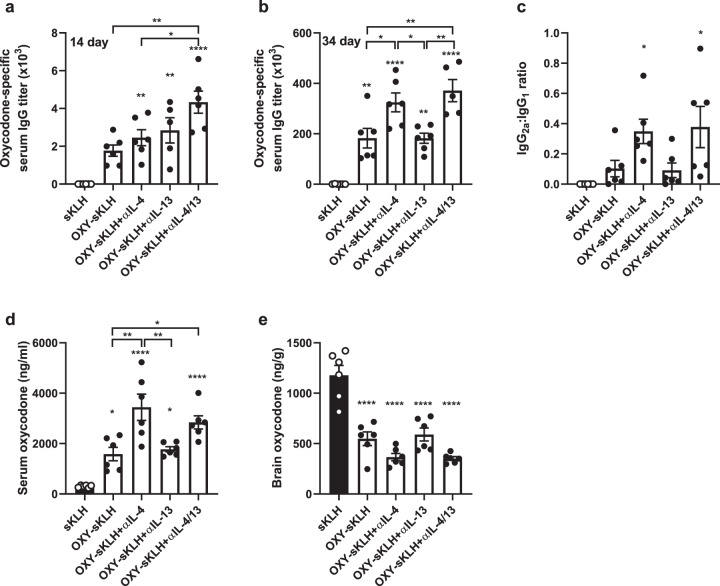
IL-13 does not modulate efficacy of anti-opioid vaccines. Balb/c mice were immunized on days 0, 14, and 28 with either sKLH or OXY-sKLH. Concurrently, αIL-4 mAb, αIL-13 mAb, or both were administered on days −2 and 1 at a dose of 0.5 mg per mAb per dose. Mice were challenged s.c. with 2.25 mg/kg oxycodone. Oxycodone-specific antibody titers expressed as: (**a**) IgG at 14 days, (**b**) IgG at 34 days, and (**c**) ratio of IgG_2a_ to IgG_1_. At 30 min post-drug challenge, concentration of oxycodone in (**d**) serum and (**e**) brain. Data are mean ± SEM. Sample size: *n* = 5–6 per group. Statistical analysis conducted by one-way ANOVA with Tukey’s multiple comparisons post hoc test. Symbols: *p ≤ 0.05, **p ≤ 0.01, ***p ≤ 0.001, ****p ≤ 0.0001 compared to control, or brackets to indicate differences between groups.

Because the IL-4R was necessary for the efficacy of OXY-KLH plus αIL-4, we hypothesized that the increased vaccine efficacy was mediated by IL-13 activating type II IL-4Rs. To test this alternative hypothesis, mice were immunized with OXY-sKLH in combination with either αIL-4, a neutralizing anti-IL-13 mAb (αIL-13), or both, to differentiate the effects of type I vs. type II IL-4R signaling. While dual IL-4/IL-13 depletion increased the early antibody response (Fig. 3a), after the third immunization, αIL-13 did not increase oxycodone-specific IgG titers compared to the vaccine alone (Fig. 3b). Furthermore, dual cytokine depletion did not have a greater effect on IgG titers than IL-4 depletion. A similar trend was found when measuring the ratio of IgG_2a_ to IgG_1_ (Fig. 3c). The lack of effect by αIL-13 was confirmed at several concentrations and dosing schedules of the αIL-13 antibody (Supplementary Fig. 2). In response to an oxycodone challenge, groups given αIL-4 showed increased serum concentrations of oxycodone (Fig. 3d) and decreased concentrations of oxycodone in the brain (Fig. 3e). Groups given αIL-13 did not display significant differences in serum or brain oxycodone concentrations when compared to the vaccine alone, and dual IL-4/IL-13 depletion did not produce any additive increases in efficacy when compared to αIL-4 administration. These data demonstrate that αIL-13 does not increase vaccine efficacy, suggesting vaccine efficacy is modulated by IL-4 signaling through the type I IL-4R. Further studies will test vaccine efficacy against opioids in IL-13R^−/−^ or IL-4/IL-4R^−/−^ mice to confirm these findings.

### Neutralization of IL-4 enhances germinal center formation after vaccination against opioids

**Fig. 4:**
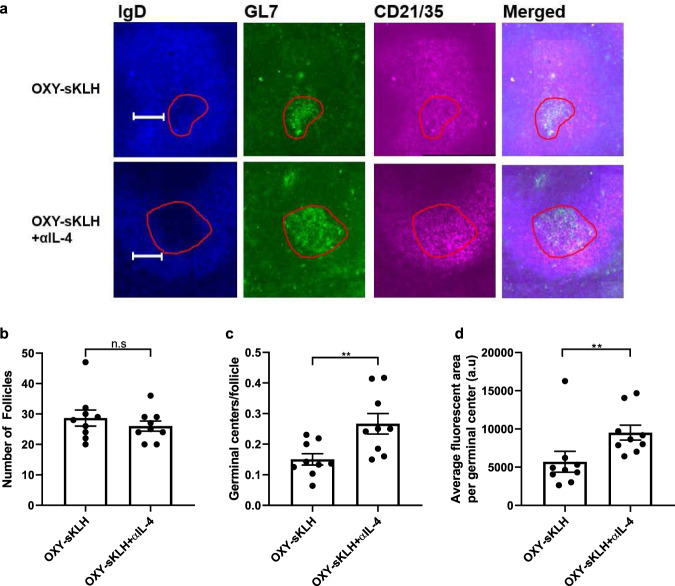
Depletion of IL-4 increases germinal center formation. Cryosections of spleens from mice vaccinated with OXY-sKLH ± αIL-4 were stained for GL7^+^ germinal center B cells, IgD^+^ follicular B cells, and CD21/35^+^ follicular dendritic cells. Slides were imaged at ×20 magnification with a Zeiss widefield fluorescence microscope. (**a**) Representative image of germinal centers and surrounding follicle from mice immunized with OXY-sKLH ± αIL-4. Germinal centers are outlined in red. At 10 days post-vaccination: (**b**) overall number of follicles per imaged spleen section, (**c**) germinal centers per follicle, and (**d**) average GL7^high^ fluorescent area per germinal center was measured. Scale bars are 100 μm. Data are mean ± SEM. Sample size: *n* = 3 mice per group × 3 slides per mouse, with slices at least 100 μm apart. Statistical analysis conducted by Mann–Whitney *U* test. Symbols: **p ≤ 0.01.

We have previously shown by means of antigen-based magnetic enrichment paired with flow cytometry in lymph nodes that the combination of OXY-KLH plus αIL-4 increased CD4^+^ T cell differentiation toward T_fh_ and GC-T_fh_ subsets, suggesting an enhancement of GC formation^40^. This study directly studied GC formation by fluorescent immunohistochemistry in murine spleens 10 days after a single immunization with OXY-sKLH with or without αIL-4. Previous findings showed that the frequency of GL7^high^ GC B cells in spleens is predictive of vaccine efficacy against oxycodone and nicotine^24,25^. Here, follicles were outlined and defined as IgD^+^CD21/35^+^ areas, which are markers of follicular B cells and follicular dendritic cells, respectively^58^. Follicular B cells developing into GC B cells downregulate expression of IgD and upregulate expression of GL7^58^, so GCs were defined as GL7^high^/IgD^low^ areas within the follicle (Fig. 4a). While the number of follicles between mice vaccinated with and without αIL-4 was not different (Fig. 4b), there was a significant increase in GCs per follicle in mice administered αIL-4 (Fig. 4c). Consistently, the GL7 positive fluorescent area was increased in αIL-4-treated mice (Fig. 4d). These data indicate that IL-4 depletion enhances anti-opioid vaccine efficacy by increasing the number and size of GCs in secondary lymphoid organs. These findings extend previous studies which demonstrated that greater efficacy of drug-conjugate vaccines against oxycodone and nicotine is correlated with increased frequency of hapten-specific GL7^high^ GC B cells in lymph nodes and spleens in mice, as measured by antigen-based magnetic enrichment paired with flow cytometry^24,25^.

### Natural Killer T (NKT) cells are not necessary for vaccine efficacy against opioids

**Fig. 5:**
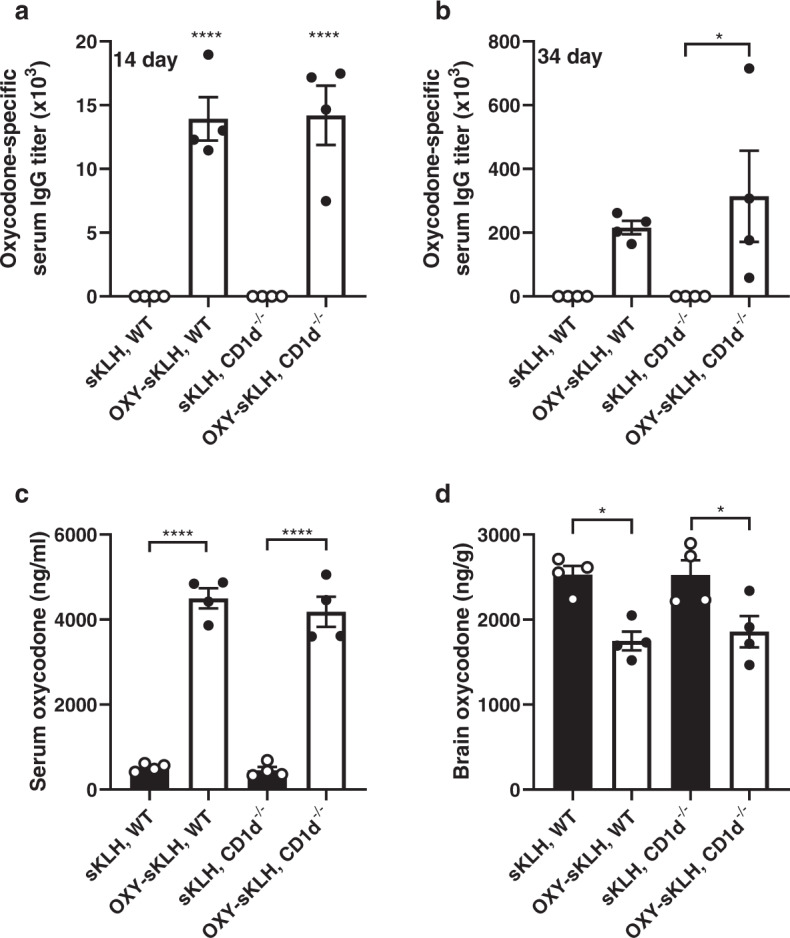
Deletion of NKT cells does not increase efficacy of anti-opioid vaccines. Wild-type or CD1d^−/−^ C57BL/6 mice were immunized with either sKLH or OXY-sKLH on days 0, 14, and 28. After immunization, oxycodone-specific antibody titers expressed as (**a**) IgG at 14 days and (**b**) IgG at 34 days. On day 35, mice were challenged s.c. with 5 mg/kg oxycodone and (**c**) serum oxycodone concentration and (**d**) brain oxycodone concentration were measured 30 min post-challenge. Data are mean ± SEM. Sample size: *n* = 4 per group. Statistical analysis conducted by one-way ANOVA with Tukey’s multiple comparisons post hoc test. Symbols: *p ≤ 0.05, ****p ≤ 0.0001 compared to control, or brackets to indicate differences between groups.

Because CD1d-restricted NKT cells are one of the main sources of IL-4 during early vaccination events^50^, we hypothesized that depletion or deletion of NKT cells would yield equivalent results to depletion or deletion of IL-4. Vaccination of wild-type and CD1d^−/−^ mice with OXY-sKLH showed no difference in oxycodone-specific IgG antibody titers after a first immunization (Fig. 5a) or after two additional boosts (Fig. 5b). In response to an oxycodone challenge, wild-type and CD1d^−/−^ mice showed no differences in serum or brain oxycodone distribution (Fig. 5c, d) suggesting that NKT cells are not necessary for successful vaccination against opioids.

### Transcriptome analysis of activated CD4^+^ T cells reveals a distinct molecular signature associated with IL-4 depletion

**Fig. 6:**
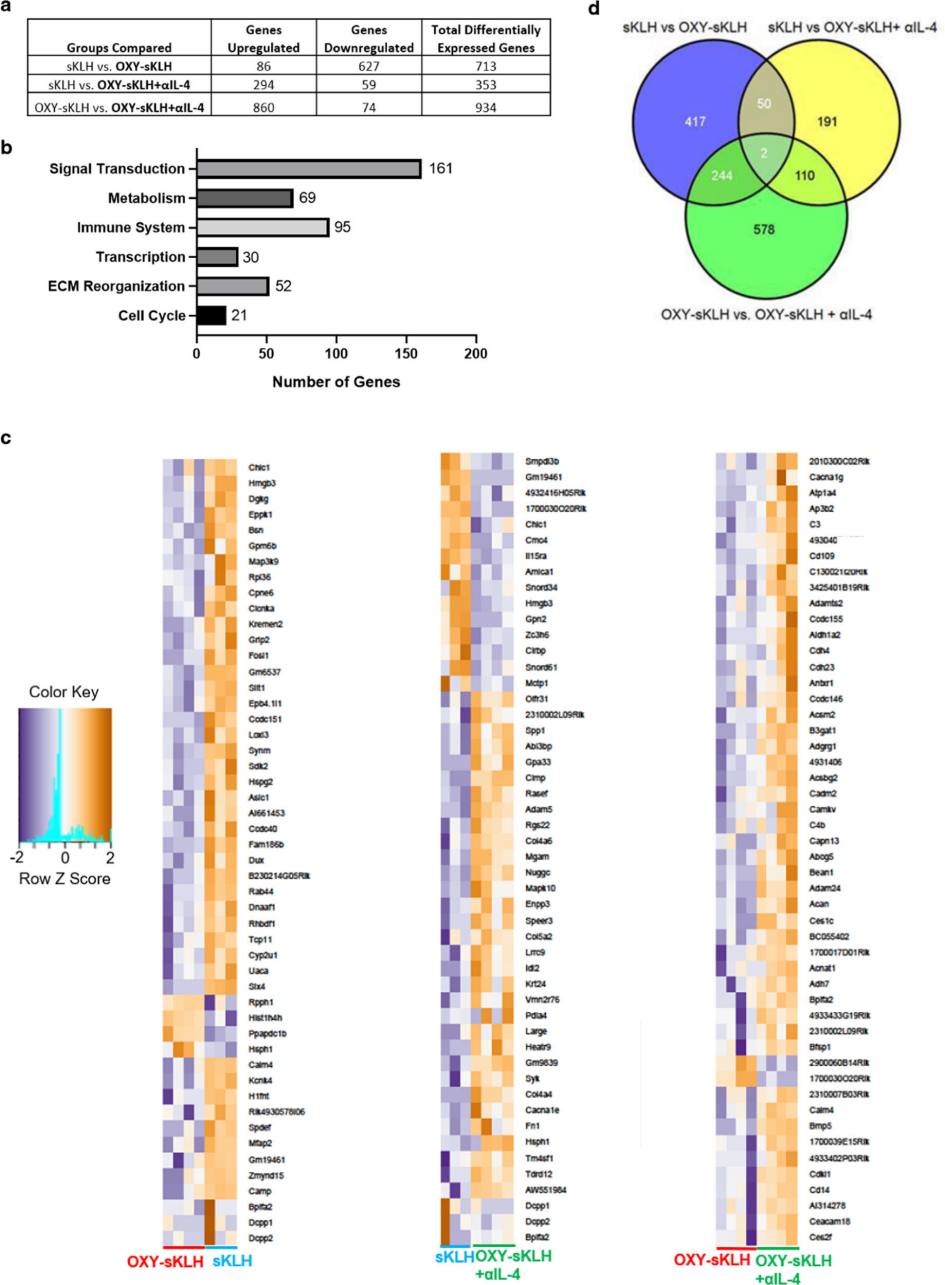
Effect of IL-4 depletion on global mRNA expression in activated CD4^+^ T cells. Mice were immunized once with either sKLH or OXY-sKLH, and αIL-4 was administered on days −2 and 1. On day 7, T cells from spleen and lymph nodes were isolated for analysis by RNA sequencing. (**a**) Table quantifying total upregulated and downregulated genes between groups. (**b**) Graph showing number of DEGs related to common cell processes between OXY-sKLH and OXY-sKLH plus αIL-4. (**c**) Comparison of top 50 upregulated and downregulated genes between sKLH vs. OXY-sKLH, sKLH vs. OXY-sKLH plus αIL-4, and OXY-sKLH vs. OXY-sKLH plus αIL-4. (**d**) Venn diagram showing similarities of DEGs between groups. Sample size: *n* = 3–4 per group. Statistical analysis conducted by edgeR on CLC Genomics Workbench for genes with at least 1.5-fold differential expression with a *p*-value ≤ 0.05 and an average of ≥5 reads per sample.

CD4^+^ T cell subset polarization is greatly influenced by IL-4 signaling^26,27,47^, consistent with our previous finding that αIL-4 skewed T cell differentiation toward GC formation^40^. To conduct a preliminary evaluation of gene expression changes associated with administration of αIL-4, RNA-sequencing was performed in sorted activated CD4^+^ T cells after immunization with OXY-sKLH with or without αIL-4 (gating strategy in Supplementary Fig. 3). When comparing all differentially regulated genes (DEGs), administration of αIL-4 led primarily to an upregulation of RNA expression (Fig. 6a). Over 500 DEGs were found to be unique to the OXY-sKLH plus αIL-4 formulation compared to the vaccine alone (Fig. 6d), which was the greatest change in gene expression when comparing any two groups to each other (sKLH control, OXY-sKLH, OXY-sKLH+αIL-4). While no specific major pathway was revealed, analysis of all 934 DEGs in the OXY-sKLH vs. OXY-sKLH+αIL-4 group suggested increased inflammatory signaling, metabolism, transcription, and signal transduction, consistent with increased T cell proliferation and differentiation triggered by immunization (Fig. 6b). The heatmap of top 50 DEGs for each group further support these findings (Fig. 6c, Supplementary Fig. 4). These data suggest that the combination of an anti-opioid vaccine and αIL-4 administration uniquely affects gene transcription in the CD4^+^ T cell compartment, supporting the need for future studies of antigen-specific CD4^+^ T cell subsets to further elucidate the cellular and molecular mechanisms associated with αIL-4-mediated enhancement of vaccines against opioids or other drug of abuse.

### Neutralization of IL-4 increases efficacy of anti-fentanyl vaccines

**Fig. 7:**
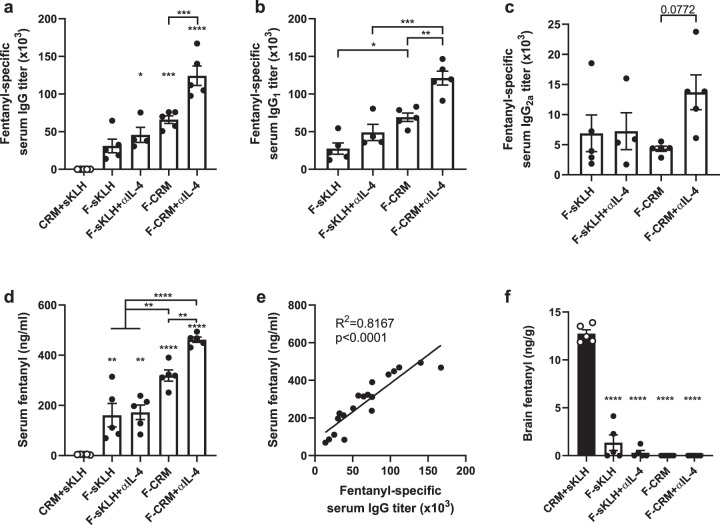
Depletion of IL-4 increases efficacy of anti-fentanyl vaccines. Balb/c mice were immunized with either F-sKLH or F-CRM, alone or in combination with αIL-4, and then challenged s.c. with 0.05 mg/kg fentanyl. Fentanyl-specific (**a**) IgG, (**b**) IgG_1_, and (**c**) IgG_2a_ subclass titers at one week after the last immunization. (**d**) Concentration of fentanyl in serum at 30 min post-drug challenge. (**e**) Linear regression of fentanyl-specific IgG titers vs. serum fentanyl concentration. (**f**) Concentration of fentanyl in the brain at 30 min post-drug challenge. Data are mean ± SEM. Sample size: *n* = 5 per group. Statistical analysis conducted by one-way ANOVA with Tukey’s multiple comparisons post hoc test. Symbols: *p ≤ 0.05, **p ≤ 0.01, ***p ≤ 0.001, ****p ≤ 0.0001 compared to control, or brackets to indicate differences between groups.

Our findings have shown that co-administration of OXY-KLH and OXY-sKLH with a depleting IL-4 antibody led to increased vaccine efficacy in preventing oxycodone-distribution to the brain, and reducing oxycodone-induced antinociception and respiratory depression^40^. To examine the applicability of this strategy to other anti-opioid vaccine formulations, a final experiment tested whether co-administration of αIL-4 would improve the efficacy of candidate anti-fentanyl vaccines containing a fentanyl-based hapten (F) conjugated to either the sKLH or CRM carrier proteins. The F-sKLH vaccine adsorbed on aluminum adjuvant is a first-generation formulation effective in preventing fentanyl distribution to the brain in mice and rats, as well as reducing fentanyl-induced respiratory depression in rats^14^. Treatment with αIL-4 increased overall IgG titers in mice immunized with F-CRM, but not F-sKLH (Fig. 7a). Mice treated with αIL-4 displayed increased IgG_1_ and IgG_2a_ titers compared to mice receiving vaccine alone (Fig. 7b, c). In response to a fentanyl challenge, F-CRM was more effective than F-sKLH in retaining fentanyl in serum, and F-CRM with αIL-4 was more effective than F-CRM alone (Fig. 7c). Serum antibody titers positively correlated with increased serum concentrations (Fig. 7d). All vaccinated groups displayed a significant reduction in brain fentanyl concentrations compared to control (Fig. 7e). The difference in effect of αIL-4 between the two vaccine formulations may relate to the different immunogenicity profiles of sKLH and CRM. Previous reports from our group showed that anti-oxycodone vaccine formulations containing either KLH or CRM were not equally effective in inducing GC B cell responses and in reducing oxycodone’s effects in mice and rats^15^. Overall, these data suggest that αIL-4 is effective in improving the efficacy of vaccines against OUD.

## Discussion

Clinical evaluation of vaccines against SUD has shown proof of efficacy in a subset of immunized patients that achieved clinically meaningful responses against the target drug of abuse^21–23^. While high levels of high affinity antibodies generally correlate with increased efficacy, it is not fully understood whether individual variability limits vaccine efficacy or whether the relatively low immunogenicity is inherent to drug-carrier conjugate vaccines. To accelerate translation, vaccine development efforts should be integrated with the analysis of underlying cellular and molecular mechanisms. A comprehensive approach would provide a blueprint for vaccine development, as well as guide personalized strategies, patient stratification, and clinical management of patients with SUD.

Previous studies have shown that the efficacy of anti-opioid vaccines could be increased by traditional adjuvant platforms, including TLR3, TLR4, TLR5, and TLR9 agonists, oil-in-water emulsions, aluminum salts, and squalene emulsions^31–34,59^. Using a previously established molecular adjuvant strategy focused on modulating the adaptive immune response^40^, this study demonstrated that αIL-4 increases efficacy of several anti-opioid vaccine formulations, suggesting that immunomodulation of IL-4 may be broadly applicable to other vaccines targeting small molecules, peptides, or purified protein subunits. These findings are consistent with reports that the depletion of IL-4 increases efficacy of vaccines against viruses in mice^55,60^. In the aforementioned studies, increased efficacy was attributed to increased IgG_1_ and IgG_2a_ production and increased numbers of cytotoxic CD8^+^ T cells. Furthermore, these studies found an increase in efficacy when blocking IL-4R and observed that IL-13 was detrimental to protective response. While we report a similar increase in antibody class switching, there was no observed effect of IL-13, and IL-4R was necessary for increased vaccine efficacy, suggesting a different mechanism than previous reports. These apparent discrepancies in the role of IL-4 in mediating vaccine efficacy against different targets may be due to inherent differences in structural and biophysical properties between antigens, such as conjugate vaccines, live attenuated viruses, or recombinant bacterial proteins. Interestingly, other studies have found that depletion of IL-4 attenuated disease progression after *Leishmania major*^61^ or *Pseudomonas aeruginosa* infection^62^. While the mechanism was not fully explored, it was postulated that this outcome was due to the change in balance between Th_2_ and Th_1_ responses. Additional studies focusing on the effects of IL-4 depletion on the CD4^+^ T cell compartment will help to determine if this mechanism plays a role in the IL-4 depletion-mediated increase in efficacy of anti-opioid vaccines.

When probing downstream signaling components of IL-4, the IL-4R was found to be required for the increase in efficacy mediated by IL-4 depletion, yet the absence of IL-13 had no effect on efficacy. These data suggest that type I IL-4R signaling plays a predominant role in modulating vaccine efficacy against drugs of abuse or small molecules. Previous studies found that genetic deletion of IL-4 similarly increased vaccine efficacy compared to pharmacologic inhibition^40^. Here, depletion of IL-4 increased vaccine efficacy yet deletion of IL-4R did not. This apparently contradictory paradigm has been observed in previous studies when examining parasitic infections in IL-4^−/−^ and IL-4R^−/−^ mice. The discrepancy was attributed to IL-13 signaling^53,63^, although this was never directly tested. In contrast, we found no contribution of IL-13, suggesting a different mechanism in the context of anti-opioid vaccines. One hypothesis is that low levels of constitutive signaling through IL-4Rs may be necessary to maintain immune system integrity after vaccination, as IL-4 signaling has been shown to prevent apoptosis in T cells and B cells^64,65^. An alternate hypothesis is that another cytokine is activating the IL-4R. To date, there is little evidence of this, however many cytokine receptors have been shown to signal through more than one ligand, including the interferon alpha receptors (IFNARs), the common gamma chain, and even the IL-4R itself^66^. Further studies using dual IL-4/IL-13 deficient mice are needed to explore whether either of these hypotheses are correct. Deletion of STAT6 also did not increase vaccine efficacy, suggesting that the effect of IL-4 after vaccination that is attenuated by depletion is not mediated through STAT6 phosphorylation. This would suggest that the increase in efficacy may be mediated through depletion of IRS1/2 signaling after Type I IL-4R signaling inhibition, although some studies have shown that the type I IL-4 receptor can also activate STAT5^67^. One caveat to these studies is the use of constitutive knockout mice which may have uncharacterized compensatory mechanisms or deficits in immune system development^68^. Studies to consolidate these seemingly divergent results of the role of IL-4 and the IL-4R in increased vaccine efficacy are an area of active investigation in our lab.

On a cellular level, αIL-4 administration increased the number and size of GCs after vaccination. Published literature shows that the depletion of IL-4 increases GC formation after secondary vaccination in some contexts^54^. However, IL-4 depletion can also have a detrimental effect on GC formation during helminth infection^44^. While the reasons behind these in vivo effects are not well established, a potential hypothesis is that both B cells and CD4^+^ T cells require progressively different cytokine environments to thrive during different stages of GC formation in response to specific antigens^42,51,69^. Accordingly, timely immunomodulation of specific cytokines (e.g., IL-4) may help to synchronize cellular and molecular events conducive to GC formation. A simpler hypothesis is that GC formation relies on a balanced CD4^+^ T cell subset polarization, and that targeted modulation of cytokines can facilitate this balance.

Our laboratory has previously shown that the generation of effective anti-drug antibodies stems from CD4^+^ T cell-dependent B cell activation, which involves GC formation^15,24^; however, the contribution of IL-4 to GC formation in the context of anti-opioid vaccination has not been fully established. To this end, this study tested whether other immune cells were involved in the effect of IL-4 blockade. Because NKT cells are one of the major sources of IL-4 after vaccination, we hypothesized that elimination of NKT cells would replicate the effect of IL-4 depletion, as it would similarly decrease IL-4 levels in the GC. However, loss of NKT cells through CD1d deletion did not influence the efficacy of anti-opioid vaccines, indicating that either these cells are not involved, or that the immune system has other compensatory mechanisms in place, such as IL-4 supplied by either T cells or basophils^70^.

On a molecular level, depletion of IL-4 resulted in a distinct transcriptomic profile in activated CD4^+^ T cells compared to the vaccine alone. While a limited number of DEGs were found, several inflammatory markers were upregulated, including complement component C3. C3 has been shown to be a general marker of inflammation^71^, an important component of antigen presentation by follicular dendritic cells^72^, and an intracellular signaling molecule in T cells which is necessary for Th_1_ responses and costimulation^73,74^. Because complement-mediated antibody effector functions are involved in neutralization of viral particles and other antigens^75,76^, it is possible that an equivalent process is involved in counteracting opioid effects, or at least in mounting an effective response to active immunization. To date, the role of antibody-mediated effector functions after vaccination against SUD is still poorly understood.

Overall, these studies are consistent with a model where the increase in anti-opioid vaccine efficacy is modulated by type I IL-4 signaling, potentially involving inhibition of the downstream insulin receptor substrate (IRS). Because the IL-4R is necessary for the increased response, these data further suggest that its basal activity in activating STAT6 or signaling through an undefined cytokine is also critical for the observed increased efficacy against oxycodone. Inhibition of IRS or other signaling may lead to increased germinal center formation in secondary lymphoid organs after vaccination, in part through changes in the T cell signaling compartment.

These results support the use of αIL-4 as a molecular adjuvant for vaccination against OUD and SUD. IL-4 depletion-mediated vaccine enhancement has now been verified with several opioid vaccine formulations, TDaP vaccines^40^, and HIV vaccines^55^. These results suggest that this strategy may be broadly applicable, particularly in vaccine formulations that require a strong neutralizing antibody response against challenging targets. Further investigation will be needed to test the cellular and molecular mechanisms underlying the modulatory effects of IL-4 on vaccine efficacy against opioids and other targets. In addition, as IL-4 polymorphisms correlated with antibody responses after vaccination against other targets^56,77^, these data support the hypothesis that IL-4 could be a putative biomarker predictive of vaccine efficacy used to identify patients that would benefit from clinical management using anti-opioid vaccines (Table 1).

**Table 1:** Summary of tested putative biomarkers predictive of vaccine efficacy against opioids.

Biomarker	Antibodies	Efficacy
Opioid-specific and hapten-specific B cells^24,25^	↑	↑
Sex (female)	↑	↑
IL-4 depletion^40^	↑	↑
IL-13 depletion	No effect	No effect
IL-2R inhibition^40^	No effect	No effect
IL-7R inhibition^40^	No effect	No effect
PD-L1 activation^40^	No effect	No effect
ICOS activation^40^	No effect	No effect
TLR3 activation^31^	↑	↑
TLR4 activation^20,34^	No effect*	No effect*
TLR9 activation^32^	↑	↑

*Sulima et al. did not directly test whether TLR4 agonists were more effective than aluminum alone.

## Methods

### Drugs

Oxycodone hydrochloride (HCl) and fentanyl citrate were obtained from either Sigma Aldrich (St. Louis, MO) or the NIDA drug supply program (RTI, NC).

### Immunomodulators

Anti-IL-4 (αIL-4) monoclonal antibody (rat anti-mouse IgG_1_, clone 11b11, Cat. No. BE0045) was obtained from Bio X cell (West Lebanon, NH). αIL-13 monoclonal antibody (mouse anti-human IgG_1_) was generously donated by Genentech (San Francisco, CA) under MTA OR-217400.

### Ethics statement

Pre-clinical studies were performed according to the Guide for the Care and Use of Laboratory Animals of the National Institutes of Health. Animal protocols were approved by the Hennepin Healthcare Research Institute and University of Minnesota Animal Care and Use Committees. Animals were euthanized by AAALAC-approved CO_2_ inhalation chambers, and efforts were made to minimize suffering.

### Mice

Six- to eight-week old male and female Balb/c and C57BL/6 mice, IL-4 receptor^−/−^ (IL-4R^−/−^, BALB/c-*Il4ra*^*tm1Sz*^/J, stock no. 003514) and STAT6^−/−^ (C.129S2-*Stat6*^*tm1Gru*^/J, stock no. 002828) mice were obtained from Jackson Laboratory (Bar Harbor, ME) or Harlan Laboratories (Madison, WI). CD1d^−/−^ mice were generously donated by Drs. Stephen Jameson and Kris Hogquist at the University of Minnesota Center for Immunology. Mice were group housed under a 12/12 h standard light/dark cycle and fed standard mouse chow ad libitum. Testing occurred during the light phase.

### Hapten synthesis and conjugate vaccines

The oxycodone-based hapten containing a tetraglycine linker at the C6 position (OXY) and the fentanyl-based hapten (F) containing an analogous linker were synthesized as previously described^14,15^, and conjugated to native keyhole limpet hemocyanin (KLH, Thermo Fisher, Rockford, IL), GMP grade subunit KLH (sKLH, Biosyn, Carlsbad, CA), or diphtheria cross reactive material (CRM, Fina Biosolutions, LLC., Rockville, MD) using carbodiimide chemistry as previously described^14,15^. For use as a coating antigen in ELISA, OXY and F haptens were conjugated to either chicken ovalbumin (OVA) or bovine serum albumin (BSA). Briefly, 5 mM OXY or F haptens were activated with 104 mM EDAC in 0.1 M MES buffer pH 5. Because of its high hydrophobicity, the F hapten was dissolved in the presence of 10% DMSO. The reaction mixture was stirred for 10 min at room temperature (RT). Native KLH, sKLH, or CRM were then added to a final concentration of 2.8 mg/mL and stirred for 3 h at room temperature. MES buffer was exchanged with PBS buffer pH 7.2 using an Amicon filter unit (MilliporeSigma, Merck, Burlington, MA) with 100 or 50 kDa molecular cutoff for sKLH and CRM conjugates, respectively, and resuspended to a final concentration of 2.5 mg/mL before storage at 4 °C. 250 mM sucrose was added as a stabilizing agent to MES and PBS buffers used for CRM conjugates. Haptenation ratio of OVA, BSA, and CRM conjugates was measured by MALDI-TOF analysis (AB SCIEX 5800, Foster City, CA), while KLH and sKLH conjugate formations were confirmed by Dynamic Light Scattering (DLS) as described^15^. The unconjugated carrier protein and the conjugate vaccines were adsorbed to aluminum adjuvant (Alhydrogel ‘85’, 2%, Brenntag Biosector, Denmark) as described in each experimental section.

### Experimental design

Anti-opioid vaccines were administered either i.m. or s.c. on days 0, 14, and 28. For studies involving the i.m route, mice were immunized in the hind leg at two sites. In all studies, mice received either the unconjugated carrier protein as a control (KLH, sKLH, or CRM) or the test vaccine (OXY-KLH, OXY-sKLH, F-sKLH, or F-CRM). In most studies, immunomodulators (αIL-4, αIL-13, or both) were administered i.p. on days −2 and 1 at a dose of 0.5 mg per injection as described^40^. Blood was collected via facial vein on days 14 and 34 for antibody analysis, unless otherwise noted. Mice were challenged s.c. on day 35 with either oxycodone (2.5–5.0 mg/kg) or fentanyl (0.05 mg/kg). Thirty minutes post-drug challenge, blood and brain were collected, processed, and analyzed for either oxycodone or fentanyl concentrations by either GC/MS as described^14^, or LC/MS. In selected experiments, vaccine efficacy in reducing opioid-induced behavioral effects was also measured by the hotplate test of centrally-mediated nociception as described^16^. Experiment-specific details are described in figure legends or individual result sections, as well as summarized in Table 2. To test the cellular and molecular mechanisms underlying vaccine efficacy, mice were immunized once, and then sacrificed at 7–10 days for either analysis of spleen sections by immunofluorescent staining or RNA sequencing in CD4^+^ T cells isolated from the spleen and lymph nodes.

**Table 2:** Experimental design.

Experiment	Treatment	Dose and route*	Adjuvant	Challenge dose
Effect of αIL-4 and sex on anti-oxycodone vaccines	sKLH, OXY-sKLH	60 μg, i.m.	Alum	5.0 mg/kg oxycodone
Test anti-oxycodone vaccines in IL-4R^−/−^ and STAT6^−/−^ mice	KLH, OXY- KLH	60 μg, s.c.	Alum	2.25 mg/kg oxycodone
Effect of αIL-4/αIL-13 on anti-oxycodone vaccines	sKLH, OXY-sKLH	60 μg, i.m.	Alum	2.25 mg/kg oxycodone
Effect of αIL-13 dosing on anti-oxycodone vaccines	sKLH, OXY-sKLH	60 μg, i.m.	Alum	2.25 mg/kg oxycodone
Effect of αIL-4 on germinal center formation (immunofluorescence)	sKLH, OXY-sKLH	60 μg, i.m.	Alum	N/A
Test anti-oxycodone vaccines in CD1d^−/−^ mice	sKLH, OXY-sKLH	60 μg, i.m.	Alum	5.0 mg/kg oxycodone
Effect of αIL-4 and anti-oxycodone vaccines on CD4^+^ T cell transcriptome by RNA-sequencing	sKLH, OXY-sKLH	75 μg, i.m.	Alum	N/A
Effect of αIL-4 on anti-fentanyl vaccines	sKLH, CRM, F-sKLH, F-CRM	50 μg, i.m.	Alum	0.05 mg/kg fentanyl

*In all experiments, αIL-4 and αIL-13 given i.p at 0.5 mg per dose, except when otherwise noted.

### Antibody analysis

Opioid-specific IgG titers were measured by ELISA as described^40^. Briefly, 96-well ELISA plates (Costar 9018 EIA/RIA, Jackson ImmunoResearch Laboratories Inc., West Grove, PA) were coated with 5 ng/well of unconjugated chicken ovalbumin (OVA) or OXY hapten conjugated to OVA in carbonate buffer at pH 9.6 overnight. For fentanyl-specific antibody titers, plates were coated with BSA, or fentanyl hapten conjugated to BSA at 5 ng/well. The following day, plates were blocked with 1% gelatin. Mouse serum was added to wells starting at a 1:200 dilution and serially diluted, and plate were incubated for 2 h. Primary antibodies were then incubated overnight with 1:30,000 goat-anti-mouse IgG (Jackson ImmunoResearch, Cat. No. 115-035-008), 1:35,000 IgG_1_ (Alpha Diagnostic International, Inc., Cat. No. 40126-GAF-BLK) or 1:7500 IgG_2a_ (Alpha Diagnostic International, Cat. No. 40127-GAF-BLK) conjugated to horseradish peroxidase to measure titers of oxycodone-specific or fentanyl-specific total IgG or individual IgG subclasses. SIGMAFAST OPD substrate (Sigma-Aldrich, St. Louis, MO) was used to develop plates.

### Drug analysis

Concentrations of oxycodone and fentanyl in brain and serum samples were determined by either single quadrupole GC/MS as described previously^14^, or by an LCMS/MS system consisting of an Agilent G6470A TQ with an Infinity II 1290 G7116B Multicolumn Thermostat, G7120A High Speed Quad Pumps, G7267B Multisampler. Data acquisition and peak integration were analyzed using Mass Hunter software (Tokyo, Japan).

### Immunofluorescent staining

Spleens were collected and immediately frozen in OCT buffer (Fisher Scientific) in a plastic cassette using liquid nitrogen and stored at −80 °C. Blocks were sliced into 10 μm sections using a cryostat, placed onto slides, and left to dry for 30 min. A hydrophobic barrier was drawn around sections using a PAP Pen (Fisher Scientific). Sections were fixed in 4% PFA for 15 min, washed, and then blocked in 5% rat serum + 0.5% IGEPAL in PBS for 1 h at room temperature. A primary antibody cocktail including 1:50 dilution GL7-FITC (Biolegend, clone GL7, Cat. No. 144603), 1:100 dilution CD21/35-PE (Biolegend, clone 7E9, Cat. No. 123409), and 1:100 dilution IgD-AF647 (Biolegend, clone 11–26 c.2a, Cat. No. 405707) in blocking solution was added to sections overnight at room temperature. Slides were then washed and one drop of Fluoroshield mounting medium with DAPI stain (Abcam) was added and a coverslip was placed on top.

### Fluorescence microscopy

Images were acquired with a Zeiss Axio.Observer Z1 motorized microscope equipped with a Plan-Apochromat ×20/0.80 NA objective and a QuantEM:512SC electron multiplying CCD camera (Photometrics). The light source was an HXP-120 mercury halide lamp and the following channels were collected sequentially (excitation/emission bands, in nm): DAPI (335–383/420–470), EGFP (450–490/500–550), phycoerythrin (559–585/600–690) and Cy5 (625–655/665–715). The large field of view was automatically tiled and stitched using ZEN 2.5 software (Zeiss). Pixel size was 800 × 800 nm. Germinal centers and follicles were quantified by manual counting by two independent counters. Counters were blinded to all treatment conditions. GL7^+^ fluorescent area was outlined and measured using Fiji software^78^.

### RNA sequencing

Spleens and lymph nodes were processed into a single cell suspension as previously described^24^. Cells were stained for viability (V450 Ghost Dye^TM^, Tonbo Biosciences, Cat. No. 13-0863-T100), CD90.2 (PerCP-eFluor710, eBioscience clone 30-H12, Cat. No. 46-0903-82), CD4 (APC-eFluor780, eBioscience clone RM4-5, Cat. No. 47-0042-82), CD44 (FITC, Biolegend clone 1M7, Cat. No. 103021), CD8a (BV510, BD Bioscience clone 53-6.7, Cat. No. 563-068), B220 (PE-Cy7, Biolegend, clone RA3-6B2, Cat. No. 103221), F4/80 (PE-Cy7, eBioscience clone BM8, Cat. No. 25-4801-82) CD11b (PE-Cy7, Biolegend clone M1/70, Cat. No. 101215) and CD11c (PE-Cy7, eBioscience clone N418, Cat. No. 25-0114-82). All antibodies were diluted 1:100, except for CD90.2, which was diluted 1:500, and the viability dye, which was diluted 1:1000. Cells were sorted for activated CD4^+^ T cells, described as Ghost Dye^−^ CD11c^−^ F4/80^−^ B220^−^ CD8a^−^ CD90.2^+^ CD4^+^ CD44^+^, using a BD FACSAria II. RNA was extracted from sorted cells using chloroform/trizol and collected using an RNA isolation kit (Qiagen, Hilden, Germany). Samples were processed using a Clontech pico mammalian RNA library preparation (Takara Bio, Mountain View, California). Libraries were sequenced on an Illumina NextSeq with 75 pair-end sequencing with samples acquiring an average of 30 million reads per sample. Results were analyzed using Reactome Pathway Analysis to analyze DEG involvement with different cellular processes^79^.

### Data analysis

Statistical analyses were performed using Prism version 8.1.2 (GraphPad, La Jolla, CA). Mean antibody titers, drug concentrations, and MPE% on the hot plate were analyzed by one-way ANOVA followed by Tukey’s multiple comparisons post hoc test. The relationship between opioid-specific antibodies, serum opioid concentrations, and brain opioid concentrations were analyzed via two-tailed Pearson correlation after determination of normality using D’Agostino-Pearson’s test. Germinal center data were analyzed using a Mann-Whitney nonparametric *U* test. In RNA-sequencing studies, data were collected and analyzed via gopher-pipelines. Briefly, 2 × 50 bp FastQ paired end reads (*n* = 33.5 Million average per sample) were trimmed using Trimmomatic (v 0.33) enabled with the optional “-q” option; 3 bp sliding-window trimming from 3’ end requiring minimum Q30. Quality control on raw sequence data for each sample were performed with FastQC. Read mapping was performed via Hisat2 (v2.1.0) using the mouse genome (mm10) as reference. Gene quantification was done via Feature Counts for raw read counts. Differentially expressed genes were identified using the edgeR (negative binomial) feature in CLCGWB (Qiagen, Redwood city, CA) using raw read counts. Statistical analysis was obtained by filtering genes on edgeR with at least 1.5-fold differential expression, a *p*-value <0.05 and a minimum average of 5 reads per group for any one gene.

### Reporting summary

Further information on research design is available in the Nature Research Reporting Summary linked to this article.

## Supplementary information

Supplementary Information

Reporting Summary

## Data Availability

All data is available upon request by contacting the corresponding author. RNA-sequencing raw data is available at NCBI under GEO repository accession number GSE157144.
