# Supplementary material for: Mechanisms of interleukin 4 mediated increase in efficacy of vaccines against opioid use disorders
Source: NPJ Vaccines. 2020 Oct 21;5:99. doi: 10.1038/s41541-020-00247-7 (PMC7578047; doi:10.1038/s41541-020-00247-7)
Supplement: Supplementary file 1 — Supplementary Information [file 41541_2020_247_MOESM1_ESM.pdf]

Mechanisms of interleukin 4 mediated increase in efficacy of vaccines against opioid use disorders

Bethany Crouse, Christine Robinson, April Huseby Kelcher, Megan Laudenbach, Juan Abrahante, and Marco Pravetoni

<u>Supplemental materials</u>

Supplemental Figure 1

Supplemental Figure 2

Supplemental Figure 3

Supplemental Figure 4

References

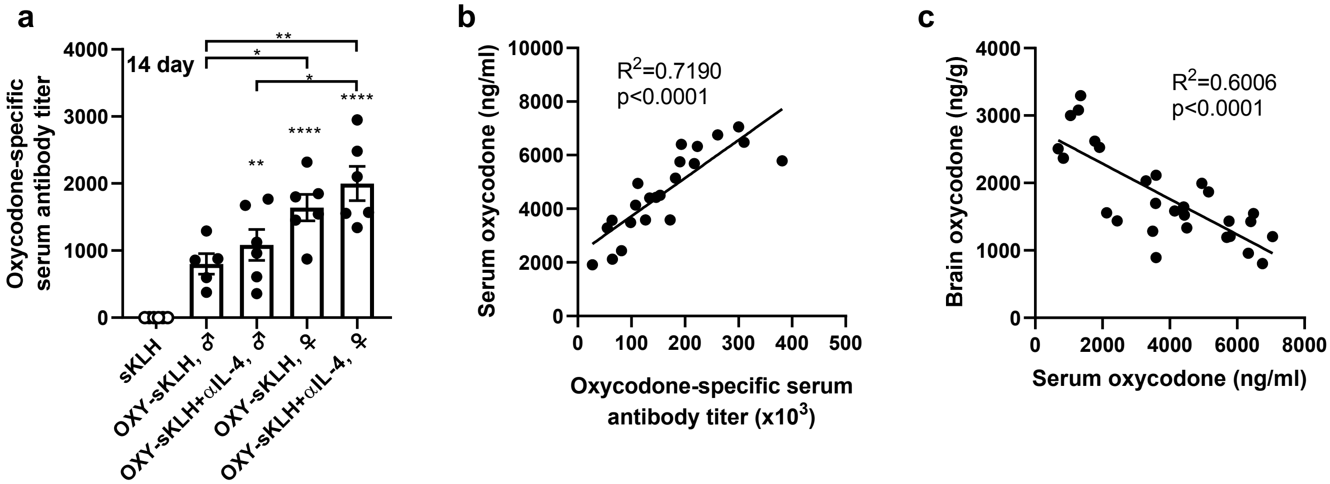

Supplemental Figure 1. Oxycodone-specific serum IgG antibody titers correlate with serum and brain oxycodone concentrations in vaccinated mice. Male and female mice were immunized with either OXY-sKLH or OXY-sKLH in combination with an anti-IL-4 mAb (αIL-4, see Figure 1) A control group received sKLH, and included both sexes. Shown: A) oxycodone-specific serum IgG titers at 14 days after the first immunization. Significant correlations were found between: B) oxycodone-specific serum IgG antibody titers and serum oxycodone concentrations, and C) serum and brain oxycodone concentrations after *in vivo* challenge with oxycodone. Data in (B) include all 4 active vaccine groups, except for sKLH. Data in (C) include all immunized mice, including the sKLH group. Data are mean±SEM. Sample size: n=5-6/group. Statistical analysis conducted by A) one-way ANOVA paired with Tukey's multiple comparisons post hoc test, and B-C) Pearson's linear regression test. Statistical symbols: * $p \leq 0.05$, ** $p \leq 0.01$, **** $p \leq 0.0001$ compared to control, or brackets to indicate differences between groups.

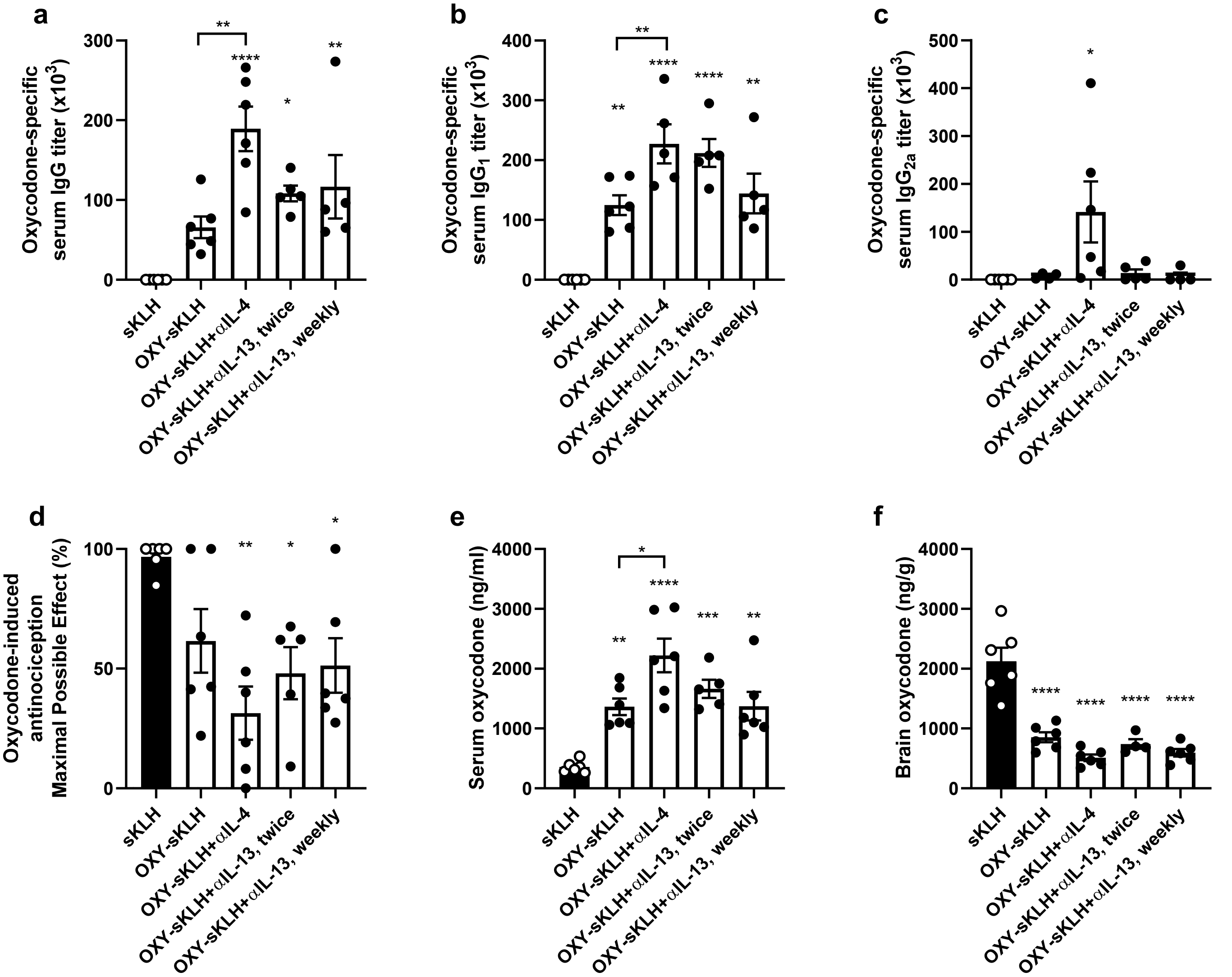

Supplemental Figure 2. Depletion of IL-13 did not alter vaccine efficacy against oxycodone. Mice were immunized on days 0, 14, and 28 with either sKLH or OXY-sKLH. Concurrently, mice immunized with OXY-sKLH were also treated with: 1) αIL-4 on days -2 and 1, 2) αIL-13 on days -2 and 1, or 3) αIL-13 once per week. αIL-4 was administered at a dose of 0.5 mg per injection as previously described [1]. αIL-13 was administered at a dose of 0.1 mg per injection based on Genentech's recommendations. After vaccination, oxycodone-specific serum antibody titers: A) IgG, B) IgG$_1$, C) IgG$_{2a}$. After *in vivo* oxycodone challenge, shown effect of vaccination on: D) decreasing oxycodone-induced antinociception percent maximum possible effect (%MPE), E) increasing opioid concentration in the serum, and F) decreasing oxycodone distribution to the brain. Data are mean±SEM. Sample size: n=5-6/group. Statistical analysis conducted by one-way ANOVA paired with Tukey's multiple comparisons post hoc test. Statistical symbols: * $p \leq 0.05$, ** $p \leq 0.01$, **** $p \leq 0.0001$ compared to control, or brackets to indicate differences between groups.

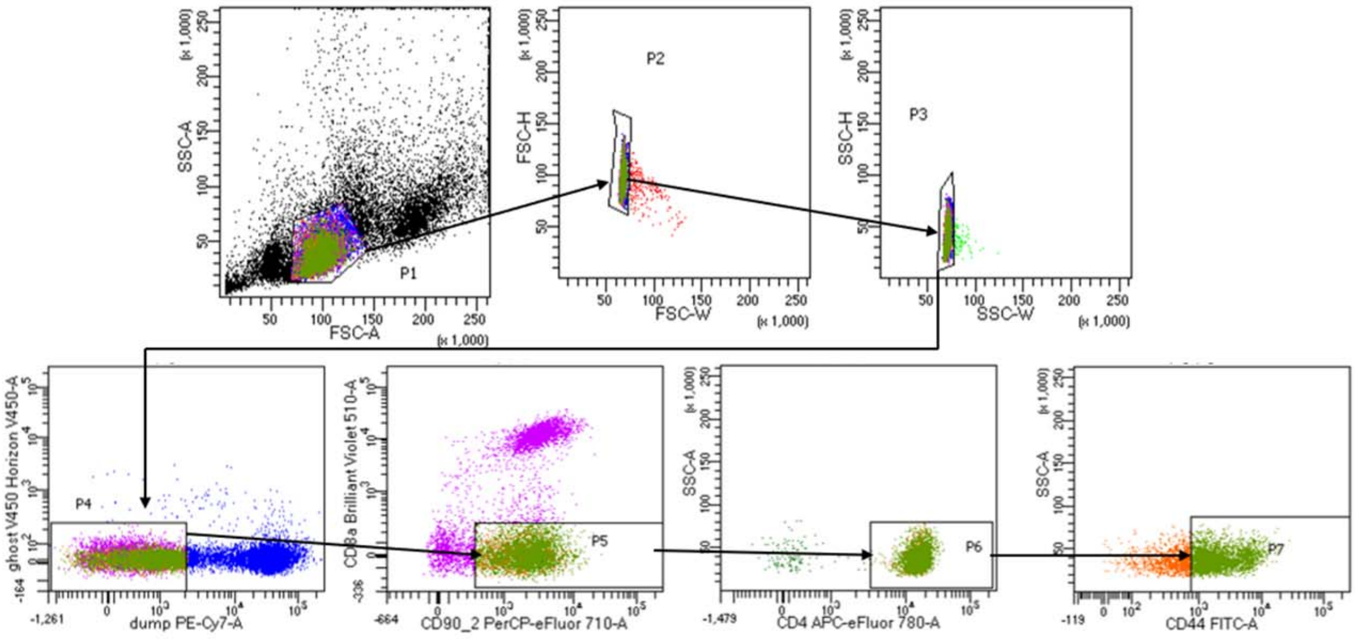

Supplemental Figure 3. Gating strategy to isolate activated CD4$^+$ T cell lymphocytes for RNA sequencing. Cells were sorted on a FACS Aria II cell sorter for live, DUMP gate negative (B220, CD11b, CD11c, F4/80), CD8a negative, CD90.2 positive, CD4 positive, CD44 positive cells as described [1].

sKLH vs. OXY-sKLH	sKLH vs. OXY-sKLH+αIL-4	OXY-sKLH vs. OXY-sKLH+αIL-4
Chic1	Smpdl3b	2010300C02Rik
Hmgb3	Gm19461	Cacna1g
Dgkg	4932416H05Rik	Atp1a4
Eppk1	1700030O20Rik	C3Ap3b2
Bsn	Chic1	4930407I10Rik
Gpm6b	Cmc4	Cd109
Map3k9	Il15ra	C130021I20Rik
Rpl36	Amica1	3425401B19Rik
Cpne6	Snord34	Adamts2
Clcnka	Hmgb3	Ccdc155
Kremen2	Gpn2	Aldh1a2
Grip2	Zc3h6	Cdh4
Fosl1	Cirbp	Cdh23
Gm6537	Snord61	Antxr1
Slit1	Mctp1	Ccdc146
Epb4.1l1	Olfr31	Acsm2
Ccdc151	2310002L09Rik	B3gat1
Loxl3	Spp1	Adgrg1
Synm	Abi3bp	4931406B18Rik
Sdk2	Gpa33	Acsbg2
Hspg2	Clmp	Cadm2
Asic1	Rasef	Camkv
AI661453	Adam5	C4b
Ccdc40	Rgs22	Capn13
Fam186b	Col4a6	Abcg5
Dux	Mgam	Bean1
B230214G05Rik	Nuggc	Adam24
Rab44	Mapk10	Acan
Dnaaf1	Enpp3	Ces1c
Rhbdf1	Speer3	BC055402
Tcp11	Col5a2	1700017D01Rik
Cyp2u1	Lrrc9	Acnat1
Uaca	Idi2	Adh7
Six4	Krt24	Bpifa2
Rpph1	Vmn2r76	4933433G19Rik
Hist1h4h	Pdia4	2310002L09Rik
Ppapdc1b	Large	Bfsp1
Hsph1	Heatr9	2900060B14Rik
Calm4	Gm9839	1700030O20Rik
Kcnk4	Syk	2310007B03Rik
H1fnt	Col4a4	Calm4
Rik4930578I06	Cacna1e	Bmp5
Spdef	Fn1	1700039E15Rik
Mfap2	Hsph1	4933402P03Rik
Gm19461	Tm4sf1	Cdkl1
Zmynd15	Tdrd12	Cd14

Camp	AW551984	AI314278
Bpifa2	Dcpp1	Ceacam18
Dcpp1	Dcpp2	Ces2f
Dcpp2	Bpifa2	

Supplemental Figure 4: Top differentially expressed genes (DEG) between treatment groups. Mice were treated with OXY-sKLH with or without IL-4 depletion (see Figure 6). Top 49-50 DEGs between treatment groups are listed as depicted in the heat map plots.

References

1. Laudenbach, M. *et al.* Blocking interleukin-4 enhances efficacy of vaccines for treatment of opioid abuse and prevention of opioid overdose. *Sci Rep* **8**, 5508 (2018).
