# Supplementary material for: Mechanisms of interleukin 4 mediated increase in efficacy of vaccines against opioid use disorders
Source: NPJ Vaccines. 2020 Oct 21;5:99. doi: 10.1038/s41541-020-00247-7 (PMC7578047; doi:10.1038/s41541-020-00247-7)
Supplement: Supplementary file 2 — Reporting Summary [file 41541_2020_247_MOESM2_ESM.pdf]

nature research

Corresponding author(s): Marco Pravetoni

Last updated by author(s): Jul 23, 2020

Reporting Summary

Nature Research wishes to improve the reproducibility of the work that we publish. This form provides structure for consistency and transparency in reporting. For further information on Nature Research policies, see our Editorial Policies and the Editorial Policy Checklist.

Statistics

For all statistical analyses, confirm that the following items are present in the figure legend, table legend, main text, or Methods section.

n/a	Confirmed	
[ ]	[x]	The exact sample size (*n*) for each experimental group/condition, given as a discrete number and unit of measurement
[ ]	[x]	A statement on whether measurements were taken from distinct samples or whether the same sample was measured repeatedly
[ ]	[x]	The statistical test(s) used AND whether they are one- or two-sided<br>*Only common tests should be described solely by name; describe more complex techniques in the Methods section.*
[x]	[ ]	A description of all covariates tested
[ ]	[x]	A description of any assumptions or corrections, such as tests of normality and adjustment for multiple comparisons
[ ]	[x]	A full description of the statistical parameters including central tendency (e.g. means) or other basic estimates (e.g. regression coefficient) AND variation (e.g. standard deviation) or associated estimates of uncertainty (e.g. confidence intervals)
[ ]	[x]	For null hypothesis testing, the test statistic (e.g. *F*, *t*, *r*) with confidence intervals, effect sizes, degrees of freedom and *P* value noted<br>*Give P values as exact values whenever suitable.*
[x]	[ ]	For Bayesian analysis, information on the choice of priors and Markov chain Monte Carlo settings
[x]	[ ]	For hierarchical and complex designs, identification of the appropriate level for tests and full reporting of outcomes
[ ]	[x]	Estimates of effect sizes (e.g. Cohen's *d*, Pearson's *r*), indicating how they were calculated

Our web collection on statistics for biologists contains articles on many of the points above.

Software and code

Policy information about availability of computer code

Data collection: *Provide a description of all commercial, open source and custom code used to collect the data in this study, specifying the version used OR state that no software was used.*

Data analysis: Statistical analyses were performed using Prism version 8.1.2 (GraphPad, La Jolla, CA)
Trimmomatic (v 0.33), Hisat2 (v2.1.0) , CLCGWB (Qiagen, Redwood city, CA) were used for RNA-sequencing studies (see description in methods)
Analysis of IHC was performed via Fiji (v2.0.0)

For manuscripts utilizing custom algorithms or software that are central to the research but not yet described in published literature, software must be made available to editors and reviewers. We strongly encourage code deposition in a community repository (e.g. GitHub). See the Nature Research guidelines for submitting code & software for further information.

Data

Policy information about availability of data

All manuscripts must include a data availability statement. This statement should provide the following information, where applicable:

- Accession codes, unique identifiers, or web links for publicly available datasets
- A list of figures that have associated raw data
- A description of any restrictions on data availability

All data is available upon request by contacting the corresponding author.

Field-specific reporting

Please select the one below that is the best fit for your research. If you are not sure, read the appropriate sections before making your selection.

- [x] Life sciences
- [ ] Behavioural & social sciences
- [ ] Ecological, evolutionary & environmental sciences

For a reference copy of the document with all sections, see nature.com/documents/nr-reporting-summary-flat.pdf

Life sciences study design

All studies must disclose on these points even when the disclosure is negative.

Sample size	In most studies, sample size was powered from our existing data to provide an ≥80% chance of detecting differences in group means with a p value of <0.05. For some experiments, number of mice was limited due to the availability and cost of transgenic mouse strains.
Data exclusions	In FIgure 2d, one data point was omitted in the STAT6-/- group, as it was determined to be an outlier via ROUT (Q=1%). This exclusion criteria was not pre-established, but we suspect was likely due to error in the machine due to previous sample contamination.
Replication	Our oxycodone and fentanyl vaccines have been extensively validated and results have been replicated in several publications, as has the increase in efficacy after IL-4 depletion. Some studies were unable to be replicated due to cost-prohibitive transgenic mouse strains, or availability of patented materials (anti-IL-13).
Randomization	In all experiments, mice were designated randomly to each treatment group.
Blinding	Researcher's were not blind to most studies, as parameters used to measure efficacy (blood/brain concentration of opioid, antibody titers) are not subject to bias. For IHC, germinal center counts were performed separately by two experimenters who were blinded to treatment conditions.

Reporting for specific materials, systems and methods

We require information from authors about some types of materials, experimental systems and methods used in many studies. Here, indicate whether each material, system or method listed is relevant to your study. If you are not sure if a list item applies to your research, read the appropriate section before selecting a response.

Materials & experimental systems

n/a	Involved in the study	
☐	☒	Antibodies
☒	☐	Eukaryotic cell lines
☒	☐	Palaeontology and archaeology
☐	☒	Animals and other organisms
☒	☐	Human research participants
☒	☐	Clinical data
☒	☐	Dual use research of concern

Methods

n/a	Involved in the study	
☒	☐	ChIP-seq
☒	☐	Flow cytometry
☒	☐	MRI-based neuroimaging

Antibodies

Antibodies used	Anti-IL-4 (αIL-4) monoclonal antibody (rat anti-mouse IgG1, clone 11b11) was obtained from Bio X cell (West Lebanon, NH). αIL-13 monoclonal antibody (mouse anti-human IgG1) was generously donated by Genentech (San Francisco, CA) under MTA OR-217400. For FACs, cells were stained for CD90.2 (PerCP-eFluor710, eBioscience clone 30-H12), CD4 (APC-eFluor780, eBioscience clone RM4-5), CD44 (FITC, Biolegend clone 1M7), CD8a (BV510, BD Bioscience clone 53-6.7), B220 (PE-Cy7, Biolegend, clone RA3-6B2), F4/80 (PE-Cy7, eBioscience clone BM8) CD11b (PE-Cy7, Biolegend clone M1/70) and CD11c (PE-Cy7, eBioscience clone N418). For IHC, sections were stained with GL7-FITC (Biolegend, clone GL7), CD21/35-PE (Biolegend, clone 7E9), and IgD-AF647 (Biolegend, clone 11-26c.2a).
Validation	Anti-IL-4: BioXcell reports clone 11b11 has <2EU/mg endotoxin (tested by LAL gel clotting assay), is >95% pure (SDS-PAGE) and is sterile filitered.They show specificity by using 11b11 as a primary antibody on a Western blot with purified mouse IL-4. They list in the reported applications as in vivo IL-4 neutralization, in vitro neutralization, in vivo IL-4 receptor stimulation (as a complex with IL-4), flow cytometry, and Western blot. Citation of this antibody is extensive and can be found at https://bxcell.com/product/m-il-4/.<br><br>Anti-IL-13: Specificity and purity of anti-IL-13 was validated by Genentech.<br><br>CD90.2 PerCP-eFluor710: Applications Tested: This 30-H12 antibody has been tested by flow cytometric analysis of mouse splenocytes<br><br>CD4 APC-eFluor780: Applications Tested: This RM4-5 antibody has been tested by flow cytometric analysis of mouse splenocytes.

CD44 FITC: Clone IM7 has been reported to recognize an epitope common to alloantigens and all isoforms of CD44 that is located between amino acids 145 and 186. This clone has been validated for immunocytochemistry (ICC) and frozen immunohistochemistry (IHC-F). Additional reported applications (for the relevant formats) include: immunohistochemistry of acetone-fixed frozen sections and formalin-fixed paraffin-embedded sections, complement-mediated cytotoxicity, immunoprecipitation, and in vivo inhibition of DTH.

CD8a BV510: Reactivity: Mouse (QC Testing); Application: Flow cytometry (Routinely Tested)

B220 PE-Cy7: Recommended Usage: Each lot of this antibody is quality control tested by immunofluorescent staining with flow cytometric analysis; Application Notes: Clone RA3-6B2 has been described to react with an epitope on the extracellular domain of the transmembrane CD45 glycoprotein which is dependent upon the expression of exon A and specific carbohydrate residues. Additional reported applications (for the relevant formats) include: immunoprecipitation, in vitro and in vivo modulation of B cell responses, and immunohistochemistry of acetone-fixed frozen sections and formalin-fixed paraffin-embedded sections.

F4/80 PE-Cy7: Applications Tested: This BM8 antibody has been tested by flow cytometric analysis of mouse resident peritoneal exudate cells.

CD11b PE-Cy7: Application: FC - Quality tested. Recommended Usage: Each lot of this antibody is quality control tested by immunofluorescent staining with flow cytometric analysis. Application Notes: Clone M1/70 has been validated for immunocytochemistry (ICC) and frozen immunohistochemistry (IHC-F).

CD11c PE-Cy7: Applications Tested: This N418 antibody has been tested by flow cytometric analysis of mouse splenocytes.

GL7 FITC: The GL-7 (GL7) antibody has been tested by flow cytometric analysis of ConA-activated mouse splenocytes. This can be used at less than or equal to 0.5 ug per test. A test is defined as the amount (ug) of antibody that will stain a cell sample in a final volume of 100 uL. Cell number should be determined empirically but can range from 10^5 to 10^8 cells/test. The GL-7 antibody has also been reported for use in immunoprecipitation and immunohistochemical staining of frozen tissue sections. It is recommended that the antibody be carefully titrated for optimal performance in the assay of interest.

CD21/35-PE: Application: FC - Quality tested. Recommended Usage: Each lot of this antibody is quality control tested by immunofluorescent staining with flow cytometric analysis.

IgD AF647: Application: FC - Quality tested. Recommended Usage: Each lot of this antibody is quality control tested by immunofluorescent staining with flow cytometric analysis. Application Notes: The 11-26c.2a antibody reacts with immunoglobulin D in all tested mouse haplotypes. The antibody binds membrane IgD expressed on most B cells. The 11-26c.2a antibody neither induces proliferation of splenic B cells nor induces B cell activation. Additional reported applications (for the relevant formats) include: immunohistochemical staining of acetone-fixed frozen sections.

Animals and other organisms

Policy information about studies involving animals; ARRIVE guidelines recommended for reporting animal research

Laboratory animals	Six- to eight-week old male and female Balb/c, C57BL/6, IL-4 receptor knockout (IL-4R-/-, BALB/c-Il4ratm1Sz/J, stock no. 003514) and STAT6-/- (C.129S2-Stat6tm1Gru/J, stock no. 002828), and CD1d-/- (B6.129S6-Del(3Cd1d2-Cd1d1)1Sbp/J Stock No: 008881) mice
Wild animals	This study did not involve wild animals
Field-collected samples	This study did not involve field-collected samples
Ethics oversight	Pre-clinical studies were performed according to the Guide for the Care and Use of Laboratory Animals of the National Institutes of Health. Animal protocols were approved by the Hennepin Healthcare Research Institute and University of Minnesota Animal Care and Use Committees.

Note that full information on the approval of the study protocol must also be provided in the manuscript.
